# Genetic Diversity of Trypanosomes Infesting Cattle from Savannah District in North of Côte d’Ivoire Using Conserved Genomic Signatures: rRNA, ITS1 and gGAPDH

**DOI:** 10.3390/pathogens13030262

**Published:** 2024-03-19

**Authors:** Jean-Yves Ekra, Eliakunda Michael Mafie, Edouard K. N’Goran, Dramane Kaba, Biégo Guillaume Gragnon, Jagan Srinivasan

**Affiliations:** 1 Department of Veterinary Microbiology, Parasitology and Biotechnology, Sokoine University of Agriculture, Morogoro 67125, Tanzania; 2 SACIDS Africa Centre of Excellence for Infectious Diseases, SACIDS Foundation for One Health, Sokoine University of Agriculture, Morogoro 67125, Tanzania; 3 Department of Biology and Biotechnology, Worcester Polytechnic Institute, 100 Institute Rd., Worcester, MA 01609, USA; 4 Unité de Formation et de Recherche (UFR) des Sciences Biologiques, Département de Biochimie-Génétique, Université Peleforo Gon Coulibaly, Korhogo BP1328, Côte d’Ivoire; ngoranedl@gmail.com; 5 Unité de Recherche «Trypanosomoses», Institut Pierre Richet 01, Bouaké BP1500, Côte d’Ivoire; kaba_dramane@yahoo.fr; 6 Laboratoire National d’Appui au Développement Agricole (LANADA), Korhogo BP1328, Côte d’Ivoire; guibiego@yahoo.fr

**Keywords:** trypanosomes, phylogenetic, cattle, Côte d’Ivoire

## Abstract

The potential danger to livestock from African animal trypanosomiasis is well known. However, the trypanosome species circulating in cattle and their genetics are poorly understood. After different alignments according to three regions (ITS1, gGAPDH and rRNA gene) of the trypanosome genome, phylogenetic analyses were used to show the genetic diversity of the different species that were circulating in the cattle in three regions (Bagoue, Poro and Tchologo) of Côte d’Ivoire. These analyses were performed by alignment of ITS1; by alignment of partial 18S, ITS1, 5.8S, ITS2 and partial 28S rRNA genes; and by alignment of gGAPDH gene with sequences of Trypanosomes found in GenBank. Three species were identified (*T. vivax*, *T. theileri* and *T. congolense*) in the cattle in the three northern regions of Côte d’Ivoire. *T. vivax* and *T. theileri* were the most abundant species in the present study. Contrary to the other primers used in this study, the ITS1 primers were not able to amplify *T. theileri*. We observed mixed infections between *T. theileri* and the other two species identified (*T. vivax* and *T. congolense*). As far as primers are concerned, in some cases, rRNA was able to identify the same species of trypanosomes that the ITS1 and gGAPDH primers were able to identify. Two main distinct groups of *T. theileri* complex were identified. The *T. congolense* and *T. vivax* strains were close to African strains, such as those from Kenya, Nigeria and Cameroon, unlike the *T. theileri* strain. Three trypanosome species (*T. vivax*, *T. theileri* and *T. congolense*) circulate in cattle in the Savannah district of Côte d’Ivoire. The genetic diversity of the trypanosome species encountered in this study cannot be classified as intraspecies according to geographical area and breed of cattle they infect.

## 1. Introduction

Trypanosomes are protozoan parasites that cause disease in humans (sleeping sickness) and cattle (Nagana) in sub-Saharan Africa [1]. In socio-economic terms, *Trypanosoma brucei*, *T. congolense* and *T. vivax* are the most important trypanosome species in sub-Saharan Africa. They are transmitted by tsetse flies (*Glossina* sp.) as a key biological vector [2]. The geographical distribution of tsetse flies is limited to sub-Saharan Africa [3].

Some species of trypanosomes can be transmitted by other vectors, such as *Trypanosoma vivax*, which can also be mechanically transmitted by *Tabanidae* and *Stomoxys* [4]. Several techniques can be used to diagnose them, but the most widely used is microscopy. Parasites are detected by optical microscopy of wet blood films or thick and thin fixed blood films stained with Giemsa [5]. Greater sensitivity can be achieved by centrifuging hematocrit to concentrate the parasite load [6]. However, these techniques do not allow the differentiation of intraspecific *Trypanosoma* spp. and are not sensitive enough to detect low levels of parasitemia [7]. Antibody detection methods, such as indirect ELISA, have been widely used in epidemiological studies.

However, these methods do not prove the existence of an ongoing infection and cannot differentiate between *Trypanosoma* spp. infections [7,8,9]. Molecular methods, notably the polymerase chain reaction (PCR), have been used to establish species-specific and interspecies detection and can detect intraspecific diversity [10]. But primers using microsatellites have shown their limitations [5]. Species-specific primers only amplify the target species and will not amplify unidentified or diverse trypanosomes that do not carry the target sequence. Other types of primers that amplify regions conserved in all trypanosome species have been developed. These include PCRs targeting the internal transcribed space (ITS) region of ribosomal genes [10,11,12]; PCRs targeting partial 18S, ITS1, 5.8S, ITS2 and partial 28S rRNA genes; and gGAPDH genes [13].

This method has been widely used due to its high sensitivity, which is attributed to a high copy number, and the feasibility of interspecies length variation allowing visual discrimination by gel electrophoresis. Amplicon sequencing can further increase sensitivity and enable intraspecific identification. These primers have been used to obtain a higher prevalence of *T. vivax* in Tanzanian tsetse populations than those based on satellite DNA sequences [13]. Thanks to these primers, new trypanosomes have been discovered in recent years. The identification of *T. simiae tsavo* was made possible by the failure of trypanosome hybridization with existing DNA probes [14]. Similarly, *T. godfreyi* was described when isoenzyme and DNA analysis indicated a trypanosome that differed from the already known species found in *Glossina morsitans submorsitans* in Gambia. Surveys of tsetse populations in Tanzania revealed a parasite that did not amplify with existing PCR primers. This led to the designation of “*T. godfreyilike*” [13] and “*T. brucei-like*” [15] parasites in tsetse flies.

In Côte d’Ivoire, trypanosome diagnostic studies using the PCR technique have been exclusively carried out with microsatellite primers [15,16,17,18]. The primers used are specific to the *T. vivax*, *T brucei* and *T. congolense* savannah and forest species. It is therefore difficult to know whether other trypanosome species exist in the natural environment of Côte d’Ivoire. Information on the genetic diversity of trypanosomes in Côte d’Ivoire is poorly known and limited. This constitutes a major weakness in the development of an effective trypanosome control strategy in Côte d’Ivoire. The lack of knowledge of this diversity constitutes a health risk for both humans and animals. In the context of One Health, it is therefore necessary to investigate the diversity of these parasites in Côte d’Ivoire. 

The objective of this work was to investigate trypanosome species diversity and their genetic diversity circulating in cattle in three regions of northern Côte d’Ivoire.

## 2. Materials and Methods

### 2.1. Sample Collection in the Field

The criteria for selecting farms and animals for blood sampling were the same as those described in our previous study [16]. Blood samples were collected from cattle in the field in a period from August to November 2021. The animals were chosen after a visual evaluation of vital indicators such as mucosal condition, respiratory rate, heart rate, pulse, body temperature, lymph nodes and animal behavior (standing position). Blood samples were prioritized for animals with low vital signs. After appropriate immobilization, a sample was taken from the jugular vein using EDTA tubes, which were kept in cold storage until the PCR analysis was performed. In addition to the blood samples, farm, and individual cattle data, such as age, breed according to the breeder, sex and body condition score, were recorded on a sample card for each herd.

### 2.2. DNA Extraction, PCR Amplification and Sequencing

DNA from blood samples was extracted using a commercially available kit (Quick-DNA™ Miniprep Plus Kit) according to the instructions of the manufacturer. 

To better understand the diversity of trypanosomes, several genome regions were selected, notably ITS1, rRNA and gGAPDH. The first time, the ITS1 region of the ribosomal RNA gene (rDNA) was amplified with ITS1 primers. The trypanosomes were identified according to their band size, as described by [11]. The reaction mixture constituted of 2 μL of template, 1X of one taq buffer, each dNTP at 200 μM, each primer at 1 μM and 0.5 U of one taq DNA polymerase (NEW ENGLAND Biolabs^R^_inc,_ Ipswich, MA, USA), adjusted at 25 μL with nuclease-free water. The cycling conditions were 94 °C for 5 min, with 35 cycles: 94 °C for 40 s, 58 °C for 40 s, 72 °C for 90 s, 72 °C for 5 min.

Secondly, the regions of the genome grouping the partial 18S, ITS1, 5.8S, ITS2 and the partial 28S were amplified by nested PCR with two pairs of ITS primers (ITS1, ITS2, ITS3 and ITS4) following the protocol described by [19]. This PCR was also performed in a 25 μL total reaction. The reaction mixtures in the first run constituted of 1X of one taq buffer, each dNTP at 200 μM, each primer (ITS1 and ITS2) at 0.4 μM, 1.25 U of one taq DNA polymerase (NEW ENGLAND Biolabs^R^_inc_) and 1 μL of DNA as template. For the second run, the first PCR product (2 μL) was added to 23 μL of a new mix composed of the same components as the first with the primers replaced by ITS3 and ITS4. The cycling conditions of both rounds were 95 °C for 7 min, with 35 cycles: 94 °C for 1 min, 55 °C for 1 min, 72 °C for 120 s, 72 °C for 5 min.

Finally, the glycosomal glyceraldehyde-3-phosphate dehydrogenase gene (gGAPDH) was amplified by nested PCR, as described by Hamilton [20], using degenerate primers (G3, G5, G4a, G1, G4b and Gs). The program was 95 °C for 3 min, with 35 cycles: 95 °C for 1 min, 55 °C for 30 s, 72 °C for 1 min and 72 °C for 10 min for both rounds. PCR products were electrophoresed in 1.5% agarose (BioTools Inc., Chuo-ku, Tokyo, Japan) in TBE buffer and stained using safe view (4 μL for 100 mL of TBE) before being visualized under UV light. The expected fragment sizes and primers sequences are shown in Supplementary Table S1.

For the sequencing of the targeted genes, the PCR products and the primers were submitted to ETON Company for sequencing by Sanger method. Afterwards, the obtained fragments were edited and aligned using CLUSTAL W program in BioEdit software to detect any conserved polymorphism between the strains. The nucleotide sequences reported in the present study are available in the DDBJ/EMBL/GenBank databases under the accession numbers presented in Table 1.

### 2.3. Analysis of Sequences

Sequence cleaning, inspection and editing were performed in BioEdit. IDs were assigned to each sequence. The IDs of the different sequences were assigned according to the following formula: The sequence ID consist of the species name, the locality abbreviation, the breed abbreviation of the infected animal, the animal number and the target genome region (Table 1). The trypanosome species corresponding to the sequences were identified by BLAST in NCBI Blastn by local alignment. Based on the three genome regions previously amplified by PCR, the alignment and comparison were only performed on the parts of the genome which were available in GenBank. Using the CLUSTAL W multiple alignment program [21] in BioEdit software, the trypanosome genome region of the ITS1 gene, gGAPDH gene and the composite region of partial 18S, ITS1, 5.8S, ITS2 and partial 28S rRNA gene nucleotide sequences were aligned with those of related trypanosomes, which were available in DDBJ/EMBL/GenBank databases. According to each region of the trypanosome genome targeted in this study, three alignments were created with trypanosome species frequently found in Africa. 

For phylogenetic analysis, rooted trees were performed. Before that, the sequences were compared at two levels: (i) between sequences obtained in the current study and available reference sequences, and (ii) between our own sequences identified as belonging to the same species or group. 

According to each region which was targeted by PCR, three neighbor-joining trees were constructed in MEGA7 [22]. The evolutionary history was inferred using maximum likelihood (ML) analysis based on the Kimura 2-parameter model (for ITS1 and gGAPDH gene) and on the Tamura 3-parameter model [23] for regions of partial 18S, ITS1, 5.8S, ITS2 and partial 28S rRNA genes. Pairwise distance estimation was performed using the Maximum Composite Likelihood (MCL) approach. The probability of inferred branches was assessed by the approximate likelihood ratio test (aLRT), an alternative to the non-parametric bootstrap estimation of branch support [24,25].

## 3. Results

### 3.1. Sequence Identification 

The present study generated 68 new sequences (Table 1). They included 4 internal transcribed spacer 1 (ITS1) sequences, 44 ribosomal DNA (18S, ITS1, 5.8S, ITS2 and 28S) sequences and 20 new sequences of the glycosomal glyceraldehyde-3-phosphate dehydrogenase gene (gGAPDH). The ITS1 sizes obtained were between 158 and 253 bp for the three sequences which shared 94.31–100% of their identity with *T. vivax* sequences found in NCBI. The 581 bp sequence was the only one that shared similarity (84%) with *Trypanosoma congolense* sequences in the NCBI database. 

For the 18S, ITS1, 5.8S, ITS2 and 28S regions, the sizes were between 550 and 1458 bp. Three and two of the 44 sequences, respectively, had a high degree of shared identity with the *T. congolense* (81.17–85.64%) and *T. vivax* (84.88–96.72%) species. The other sequences were very close to the *T. theileri* species, with shared identities of between 83.02 and 99.43%.

For the gGAPDH gene, we had eight sequences that were close to the *T. vivax* species with 84.55–90.83% similarity in identity, ranging in size from 300 to 752 bp. Eleven (11) sequences were identified as very close to *T. theileri*, with identity percentages between 91.23 and 97.98%. Of the twenty sequences obtained based on this region of the trypanosome genome, only one sequence was identified as being very close to the *T. congolense* species, with an identity similarity of 90.08%, with the accession number AJ620289.1 as reference.

### 3.2. Mixed Infection and Mixed Identification by Primers

In this study, we obtained mixed infections from animals and species infection cases that were identified simultaneously by at least two of the types of primers used. This can be seen in Table 1, so the IDs with the same letter in the superscripts labeled in red are cases of mixed infection, and the IDs with the same number in the superscripts written in blue are cases of species identified by at least two of the primer types in this study.

For mixed infections, we had two cases of *T. theileri*/*T. vivax* couplings and one case of a *T. theileri/T. congolense* coupling. As examples, we list the following IDS: *T. theileri_Fer_M_362_RNA* (*OR973760*) and *T. vivax_Ferk_M_362* (*PP210927*), as well as *T. theileri_Mben_M_272_RNA* (*PP188050*) and *T. congo_Mben_M_272_RNA* (*PP188052*).

In the case of trypanosome species identified by more than one primer, we observed two possibilities in this study: ITS1/rRNA coupling or GAPDH/rRNA coupling. We obtained one case with an ITS1/rRNA coupling (*T. congo_Mben_M_271* (*PP210925*) and *T. congo_Mben_M_271_RNA* (*PP188049*)), which enabled us to identify the same strain of *T. congolense*. The GAPDH/rRNA pairing enabled us to identify seven *T. vivax* strains (*T. vivax_Diko_N_225_RNA* (*PP188048*) and *T. vivax_Diko_N_225_GAPDH* (OR966685)) and six *T. theileri* strains (Table 1).

### 3.3. Sequence Comparison between the Different Trypanosome Species Identified

Of the ITS1 nucleotide sequences of *T. vivax* (Supplementary Figure S1), the different individuals identified had 245 nucleotides in common, corresponding to a similarity of 90.40%. We found 16 insertions/deletions (Indel) and 10 nucleotide substitutions. As for the *T. vivax* GAPDH gene sequence, there was a 30% similarity (90 matches) between the sequences, 130 nucleotide substitutions and 136 insertions/deletions (Indel) when all sequences were considered simultaneously.

Nucleotide sequences covering the partial 18S, ITS1, 5.8S, ITS2 and partial 28S rRNA gene regions of *T. theileri* shared a similarity of 27.43% (203 nucleotides), with 309 insertions/deletions (Indel) and 381 nucleotide substitutions between all these individuals. For the *T. theileri* GAPDH gene sequences, there was 67.50% similarity, which corresponds to 162 nucleotide matches between all these sequences, 84 nucleotide substitutions and 6 insertions/deletions (Indel), when comparing all sequences simultaneously (Supplementary Figure S2).

Comparing the *T. congolense* sequences with reference to the partial 18S, ITS1, 5.8S, ITS2 and partial 28S rRNA genes, we observed 36 insertions/deletions (Indel), 90 nucleotide substitution and 239 identical nucleotides; this represents 68.480% similarity between the three *T. congolense* genotypes identified (Supplementary Figure S3).

### 3.4. Alignment and Phylogenetics of Trypanosome Species According to Internal Transcribed Spacer 1 (ITS1)

**Figure 1:**
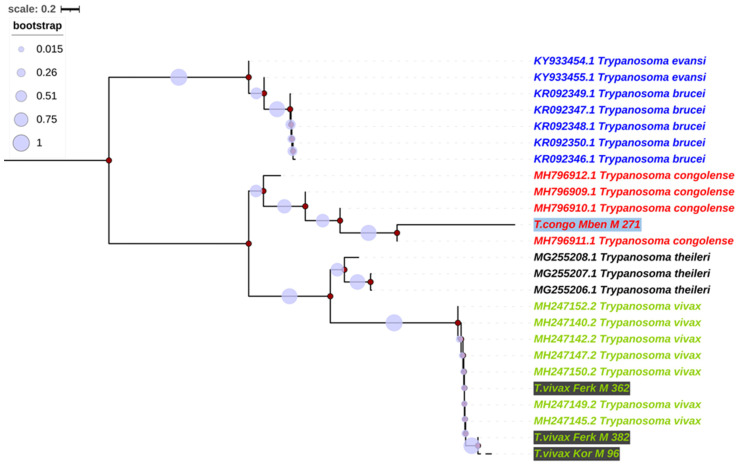
Phylogenetic analysis based on internal transcribed spacer 1 (ITS1). The nucleotide sequences of *T. vivax* described in this study are written in green with a gray background, and the nucleotide sequences of *T. congolense* are written in red with a blue background. Evolutionary history was inferred using the neighbor-joining method [26]. The percentages of replicate trees in which the associated taxa clustered together in the bootstrap test (1000 replicates) are shown next to the branches [27]. The tree is drawn to scale, with branch lengths in the same units as those of the evolutionary distances used to infer the phylogenetic tree. The evolutionary distances were computed using the Maximum Composite Likelihood method [28] and are in the units of the number of base substitutions per site. Evolutionary analyses were conducted in MEGA7 [22] and iTOL (https://itol.embl.de/itol.cgi; accessed on 23 February 2024).

The internal transcribed spacer 1 (ITS1) alignment was used to construct phylogenetic trees using neighbor joining, a maximum likelihood method based on the Kimura two-parameter model [25]. The ITS1 sequences of this study clustered with different reference sequences (Figure 1), and three sequences formed strong groups with *T. vivax* sequences from the NCBI database. This group formed a separate clade (bootstrap value: 90%) with a clade formed by *T. congolense*, *T. theileri*, *T. brucei* and *T. evansi*. One ITS1 sequence of this study was identified as a *T. congolense* sequence. This sequence is part of the clade of *T. congolense* with a bootstrap of 60% (Figure 1).

### 3.5. Phylogenetic Trees of Trypanosomes Species According to Partial 18S, ITS1, 5.8S, ITS2 and Partial 28S rRNA Genes

Based on the alignment of the partial 18S, ITS1, 5.8S, ITS2 and partial 28S rRNA genes for the sequences generated in this study with those sequences from the NCIBI, a phylogenetic tree was constructed. The distances between the different sequences were estimated using the Maximum Composite Likelihood (MCL). 

**Figure 2:**
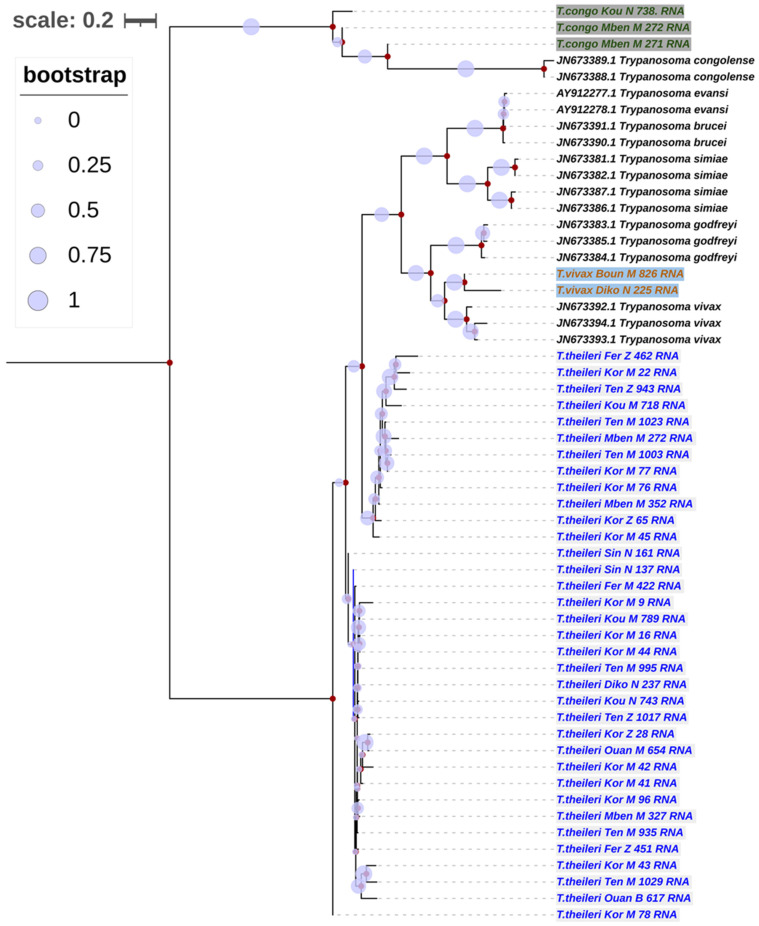
Phylogenetic analysis based on partial 18S, ITS1, 5.8S, ITS2 and partial 28S rRNA genes. The nucleotide sequences described in this study are written in green, orange and blue, respectively, with a background for *T. vivax*, *T. congolense* and *T. theileri* species. Evolutionary history was inferred using the neighbor-joining method [26]. The percentages of replicate trees in which the associated taxa clustered together in the bootstrap test (1000 replicates) are shown next to the branches [27]. The evolutionary distances were computed using the Maximum Composite Likelihood method [28] and are in the units of the number of base substitutions per site. The rate of variation among sites was modeled with a gamma distribution (shape parameter = 3). All ambiguous positions were removed for each sequence pair. Evolutionary analyses were conducted in MEGA7 [22] and iTOL (https://itol.embl.de/itol.cgi; accessed on 23 February 2024).

The sequences of *T. vivax* (2 sequences), *T. congolense* (3 sequences) and *T. theileri* (39 sequences) species were identified as close to the sequences obtained in the current study. The alignment from the partial 18S, ITS1, 5.8S, ITS2 and partial 28S rRNA genes for the sequence generated showed a phylogenetic tree with two main groups of *T. theileri* (Figure 2). The first group comprised 12/39 sequences, and the second group had 29/39 sequences. The two large, distinct *T. theileri* groups formed by our sequences had a bootstrap value of 57% (Figure 2). The two sequences of *T. vivax* formed a strong clade with those from the NCBI database. This *T. vivax* clade was part of a big clade constituted of clades formed by *T. godfreyi*, *T. simiae*, *T. brucei* and *T. evansi*, which are more closely related to the *T. theileri* taxon 1, with a bootstrap value of 79% (Figure 2). 

### 3.6. Phylogenetic Trees of Trypanosome Species According to Glycosomal Glyceraldehyde-3-Phosphate Dehydrogenase Gene (gGAPDH)

The Maximum Composite Likelihood (MCL) approach allowed us to estimate the distance between the sequences of this study and those from the NCIBI GenBank.

**Figure 3:**
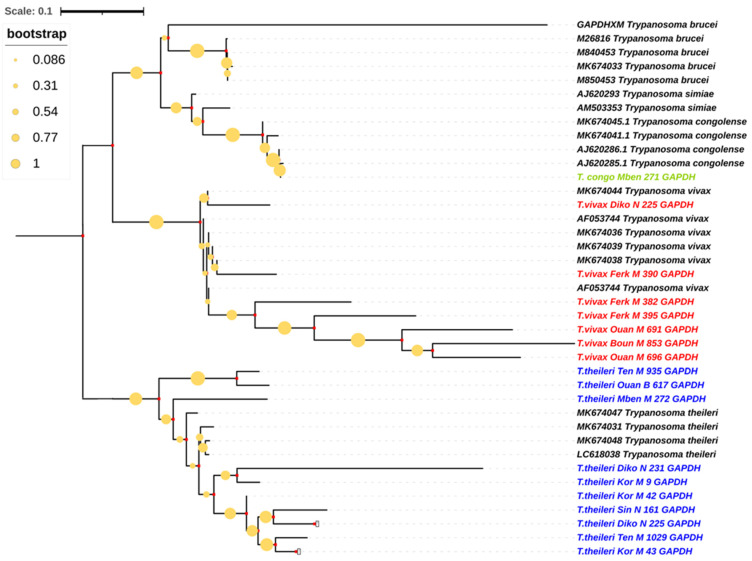
Phylogenetic analysis based on glycosomal glyceraldehyde-3-phosphate dehydrogenase gene (gGAPDH). The nucleotide sequences described in this study are written in green, red and blue, respectively, for *T. vivax*, *T. congolense* and *T. theileri* species. Evolutionary history was inferred using the neighbor-joining method [26]. The percentage of replicate trees in which the associated taxa clustered together in the bootstrap test (1000 replicates) are shown next to the branches [27]. The evolutionary distances were computed using the Maximum Composite Likelihood method [28] and are in the units of the number of base substitutions per site. All ambiguous positions were removed for each sequence pair. Evolutionary analyses were conducted in MEGA7 [22] and iTOL (https://itol.embl.de/itol.cgi; accessed on 25 February 2024).

Sequences identified as *T. vivax*, *T. theileri* and *T. congolense* had strongly constituted clades according to their reference’s sequences. Here, two clades formed in the generated sequences, the *T. vivax* clade and the big clade formed by the *T. theileri* clade, the *T. brucei* clade and the *T. congolense* clade. The *T. vivax* clade formed a single clade which was very distinct, with a 100% bootstrap value. The *T. congolense* sequence was closer to the *T. theileri* clade than to the one of the *T. vivax* clade (Figure 3).

## 4. Discussion

Analysis of PCR product sequences according to three different regions of the trypanosome genome obtained from the blood of cattle collected in the Bagoue, Tchologo and Poro regions has enabled us to identify three trypanosome species: *T. vivax*, *T. congolense* and *T. theileri*.

The ITS1 primers were unable to identify the *T. theileri* species. The ability of ITS1 primers to detect more *T. vivax* and *T. congolense* species and fewer *T. theileri* was demonstrated by Njiru et al. [11]. Indeed, the ITS1 primer was designed to improve the detection of pathogenic trypanosome species by minimizing its homology with non-pathogenic species, notably *T. theileri*, which is non-pathogenic [5]. This could be because during primer design, it may not have been considered due to its non-pathogenic nature.

Primers amplifying the gGAPDH region and the one amplifying the partial 18S, ITS1, 5.8S, ITS2 and partial 28S rRNA genes were used to identify the three species encountered in this study. In contrast, the prevalence of *T. congolense* and *T. theileri* species obtained with primers amplifying the partial 18S, ITS1, 5.8S, ITS2 and partial 28S rRNA genes was high compared to their prevalence with gGAPDH. This could be explained by the presence of more copies of partial 18S, ITS1, 5.8S, ITS2 and partial 28S rRNA genes than copies of the gGAPDH gene. It is important to note that the situation was different for the *T. vivax* species, whose high prevalence was revealed by the gGAPDH. This low prevalence of *T. vivax* according to partial 18S, ITS1, 5.8S, ITS2 and partial 28S rRNA genes was also observed by Auty et al. [29], who used a different primer for *T. vivax* amplification. This could be explained by a problem specific to this region of the *T. vivax* species.

Here, we have a sensitivity and specificity of primers that would relatively depend on the target region and the trypanosome species in question. 

In this study, we noted more *T. vivax* sequences than *T. congolense* sequences, which was also noted by other researchers [16,17,30]. *T vivax* and *T. theileri* were present in mechanical vectors, notably *Tabanidae*. The present study area, in this case the savannah district, is a predilection zone for *Tabanidae* [31]. Several researchers [29,30,31] have demonstrated the role of *Tabanidae* in the transmission of *T. vivax*. The work of [31] showed the dispersion and abundance of *Tabanidae* in the different ecological facies of this study area. Twelve (12) species were identified, with *Tabanus laverani* being the most encountered species with an ADP of 906 individuals/trap/day. The *Tabanidae* species are also potential vectors of the *T. theileri* species [4,32,33]. Kostygov et al. [34] demonstrated the life cycle of two different species of the *Trypanosoma theileri* complex in *Tabanidae*. The strong presence of *T. vivax* and *T. theileri* species are therefore closely linked to the strong presence of the *Tabanidae* vectors of these two species, whose population is high in this area [29,32]. 

Comparison between the sequences of individuals gave us 68.48%, 27.43% and 90.40% similarity between the nucleotide sequences of *T. congolense*, *T. theileri* and *T. vivax* species, respectively, considering the portions including the ITS1 part. The 27.43% similarity between *T. theileri* sequences was low. This may be due to the high number of sequences compared at the *T. theileri* species level. According to keita [20,35], sequence comparison is a function of sequence number and this has an impact on the identity rate. When the datasets are small enough, very precise alignments may be calculated. Alignments of huge datasets that have developed with numerous indels will most likely have high error rates, and gene trees constructed from these alignments will similarly have significant errors [36].

We a high diversity of the *T. theileri* complex. The blast results showed that the strains were close to certain sources from horizons that are geographically far from the present study area, such as North America, South America, Asia and Europe. This could be explained by the fact that the gGAPDH and rRNA regions of the *T. theileri* species are so conserved that it is impossible to distinguish between those from Africa and other continents. On the other hand, it may be due to the low availability of sequences from African strains of this part of the *T. theileri* genome in the NCBI database. Given the low pathogenicity of this species, it has been neglected by African researchers. In fact, they have made greater use of specific primers and have been more focused on three species: *T. brucei*, *T. congolense* and *T. vivax* [37]. By observing the phylogenetic trees according to the rRNA gene we were able to classify the *T. theileri* complex into two distinct main groups. These observations are in line with those of Kostygov [34], who was able to show that two different species of the *T. theileri* complex, for which we had no prior information on the morphology of their blood forms, belong to two different clades of the complex, named *TthI* and *TthII*. However, these species have not been distinguished by morphology, morphometry, ultrastructure or development in the vector. Similarly, this distinction of the *T. theileri* species into two different clades is not possible when considering the geographical areas and the hosts of the *T. theileri* species to which they are genetically close according to phylogenetic trees.

The *T. congolense* species observed in this study are closely related to strains from the greater West African zone, more specifically Nigeria and Cameroon. The phylogenetic proximity of the *T. congolense* populations observed in this study can be broken down into two subspecies. The strain obtained in the Kouto department is genetically close to the savannah-type *T. congolense* subspecies. The strains obtained in the M’Bengue area are genetically close to the forest-type *T. congolense* subspecies. In fact, *T. congolense* species belong to the *Nannomonas* genus, which has been the subject of much morphological and genetic research. Currently, the recognized genotypes of the *nannomonas* genus are savannah-type *T. congolense*, West African forest/river-type *T. congolense* and Kilifi-type *T. congolense* [13,35,38].

According to the gGAPDH gene, we found 30% similarity between *T. vivax* individuals and 67.50% at the *T. theileri* level despite the high number of individuals compared at *T. theileri* level. This observation demonstrates the high diversity found within the trypanosomes of the *T. vivax* species. This high diversity of the *T. vivax* species could be linked to the large number of vectors it has, notably mechanical vectors (*Tabanidae*, *Stomoxes*) and tsetse flies [39]. In fact, this species’ ability to spend a stage of its cycle in the digestive tract of different vectors may play a role in the perpetual modification of its genetic material. According to the present phylogenetic tree, the *Trypanosoma vivax* species encountered in this study are very close to the Kenyan strains, based on the ITS1 part of the genome. On the other hand, based on the gGAPDH gene, the *T. vivax* species are close to those from Cameroon (Central Africa). According to Adams [40], it would be better to classify *T. vivax* variants into types A, B and C on the basis of genetic variants. So, in the present case, based on the ITS1 region, the strains are types A or B, but they are type C according to the gGAPDH gene. It is important to note that the geographical nomenclature used in previous works to name trypanosomes can be misleading. Clearly, taking genetic data into account in historical taxonomic classifications is not an easy task. The nomenclature suggested by Adams [40] for naming groups A, B and C should be further developed, taking into account variations on the part of the *T. vivax* genome by comparing more strains from different parts of Africa based on different parts of the genome, like the small subunit ribosomal RNA gene (SSU rDNA), the mitochondrial DNA or the kinetoplast DNA (minicircles and maxicircles). 

## 5. Conclusions

Analysis of the ITS1, rRNA and gGAPDH regions of trypanosomes circulating in cattle in the Savannah district of Côte d’Ivoire identified three trypanosome species, including a species that had been neglected (*T. theileri*) in terms of study in Côte d’Ivoire as well as certain species that are important as livestock pathogens (*T. vivax* and *T. congolense*). The observed variability in these three strains cannot be classified according to the geographical area and breed of cattle they infect. In addition, due to the fact that livestock farming has been recognized for many years as being under pressure from pathogens originating from a variety of vectors, the addition of phylogenetic information raises many questions about livestock trypanosomes, particularly regarding transmission, host sharing and pathogenicity. This is important, as livestock morbidity and mortality due to *T. congolense* and *T. vivax* infections continues to cause heavy losses to livestock farmers. These strains should be studied in other domestic animals (sheep, goats, etc.), as well as in wild animals. Further phylogenetic analysis will probably be needed to explore these complex relationships.

## Figures and Tables

**Table 1:** Sequences of trypanosomes identified in this study.

Sequence ID	Accession Number	Bovine Race and Location	Length of Sequence (bp)	Similarity (%)	Reference Sequence Accession	Origin Country of Reference
		ITS1				
*T.vivax_Kor_M_96 **^a^***	PP210924	Méré/Korhogo	209	97.56	MH247152.2 *T. vivax*	Kenya
*T.congo_Mben_M_271* **^1^**	PP210925	Méré/Mbengue	666	80.57	MK756202.1 *T. congolense*	Nigeria
*T.vivax_Ferk_M_382*	PP210926	Méré/Ferkéssédougou	250	94.31	MN213748.1 *T. vivax*	Sub-Saharan Africa
*T.vivax_Ferk_M_362 **^b^***	PP210927	Méré/Ferkéssédougou	250	100	MN213748.1 *T. vivax*	Sub-Saharan Africa
		18S, ITS1, 5.8S, ITS2, 28S				
*T.theileri_Kor_M_9_RNA* **^2^**	PP188043	Méré/Korhogo	932	92.60	LC440407.1 *T. theileri*	Mongolia
*T.theileri_Kor_M_16_RNA*	PP188044	Méré/Korhogo	939	99.43	OQ341207.1 *T. theileri*	Ecuador
*T.theileri_Kor_M_22_RNA*	OR973744	Méré/Korhogo	707	84.18	LC440405.1 *T. theileri*	Mongolia
*T.theileri_Kor_Z_28_RNA*	OR973745	Zébu/Korhogo	872	94.13	OQ341206.1 *T. theileri*	Ecuador
*T.theileri_Kor_M_41_RNA*	PP188045	Méré/Korhogo	930	93.87	LC440408.1 *T. theileri*	Mongolia
*T.theileri_Kor_M_42_RNA* **^5^**	OR973746	Méré/Korhogo	776	92.22	AB569248.1 *T. theileri*	Japan
*T.theileri_Kor_M_43_RNA* **^6^**	OR973747	Méré/Korhogo	957	90.27	KY412803.1 *T. theileri*	Austria
*T.theileri_Kor_M_44_RNA*	OR973748	Méré/Korhogo	912	99.01	OQ341206.1 *T. theileri*	Ecuador
*T.theileri_Kor_M_45_RNA*	PP188046	Méré/Korhogo	939	98.73	LC440405.1 *T. theileri*	Mongolia
*T.theileri_Kor_Z_52_RNA*	OR973749	Zébu/Korhogo	930	99.45	OQ341205.1 *T. theileri*	Ecuador
*T.theileri_Kor_Z_65_RNA*	OR973750	Zébu/Korhogo	744	90.87	OQ341215.1 *T. theileri*	Ecuador
*T.theileri_Kor_M_76_RNA*	OR973751	Méré/Korhogo	764	94.89	AB569249.1 *T. theileri*	Japan
*T.theileri_Kor_M_77_RNA*	OR973752	Méré/Korhogo	939	97.23	AB569249.1 *T. theileri*	Japan
*T.theileri_Kor_M_78_RNA*	OR973753	Méré/Korhogo	687	88.35	AB569248.1 *T. theileri*	Japan
*T.theileri_Kor_M_96_RNA **^a^***	OR973754	Méré/Korhogo	914	98.57	KY412803.1 *T. theileri*	Austria
*T.theileri_Sin_N_119_RNA*	OR973755	N’Dama/Sinématiali	617	88.77	OQ341215.1 *T. theileri*	Ecuador
*T.theileri_Sin_N_137_RNA*	OR973756	N’Dama/Sinématiali	716	99.16	LC440408.1 *T. theileri*	Mongolia
*T.theileri_Sin_N_161_RNA*	PP188047	N’Dama/Sinématiali	931	98.83	OQ341205.1 *T. theileri*	Ecuador
*T.vivax_Diko_N_225_RNA* **^3^**	PP188048	N’Dama/Dikodougou	575	84.88	KM391836.1 *T. vivax*	Nigeria
* T.theileri_Diko_N_237_RNA*	OR973757	N’Dama/Dikodougou	925	98.26	OQ341205.1 *T. theileri*	Ecuador
*T.congo_Mben_M_271_RNA* **^1^**	PP188049	Méré/M’Bengué	815	85.40	U22319.1 *T. congolense*	Ecuador
*T.theileri_Mben_M_272_RNA* ***^c^***	PP188050	Méré/M’Bengué	918	93.69	OQ341215.1 *T. theileri*	Ecuador
*T.congo_Mben_M_272_RNA* ***^c^*** **^9^**	PP188052	Méré/M’Bengué	710	85.76	U22319.1 *T. congolense*	Kenya
* T.theileri_Mben_M_327_RNA*	OR973758	Méré/M’Bengué	819	97.68	LC440408.1 *T. theileri*	Ecuador
*T.theileri_Mben_M_352_RNA*	OR973759	Méré/M’Bengué	750	96.55	AB569249.1 *T. theileri*	Japan
*T.theileri_Fer_M_362_RNA* ***^b^***	OR973760	Méré/Ferkéssédougou	708	95.28	AB569249.1 *T. theileri*	Japan
* T.theileri_Fer_M_422_RNA*	OR973761	Méré/Ferkéssédougou	757	97.74	OQ341209.1 *T. theileri*	Ecuador
*T.theileri_Fer_Z_451_RNA*	OR973762	Zébu/Ferkéssédougou	904	96.50	KY412803.1 *T. theileri*	Austria
* T.theileri_Fer_Z_462_RNA*	OR973763	Zébu/Ferkéssédougou	763	85.87	LC440405.1 *T. theileri*	Mongolia
*T.theileri_Ouan_B_617_RNA* **^4^**	OR973764	Baoulé/Ouangolodougou	700	87.43	JX178185.1 *T. theileri*	USA
*T.theileri_Ouan_M_654_RNA*	OR973765	Méré/Ouangolodougou	880	95.72	OQ341206.1 *T. theileri*	Ecuador
* T.theileri_Kou_M_718_RNA*	OR973766	Méré/Kouto	931	89.04	AB569249.1 *T. theileri*	Japan
*T.congo_Kou_N_738_RNA*	PP188051	N’Dama/Kouto	1457	85.64	U22319.1 *T. congolense*	Ecuador
*T.theileri_Kou_N_743_RNA*	OR973767	N’Dama/Kouto	934	98.93	OQ341206.1 *T. theileri*	Ecuador
*T.theileri_Kou_M_789_RNA*	OR973768	Méré/Kouto	924	99.14	KY412803.1 *T. theileri*	Austria
*T.vivax_Boun_M_826_RNA*	PP188053	Méré/Boundiali	549	96.72	KC196660.1 *T. vivax*	Gambia
*T.vivax_Boun_M_854_RNA*	OR973769	Méré/Boundiali	740	83.02	AB569249.1 *T. theileri*	Japan
* T.theileri_Ten_M_935_RNA*	OR973770	Méré/Tengréla	922	99.23	KY412803.1 *T. theileri*	Austria
* T.theileri_Ten_Z_943_RNA*	OR973771	Zébu/Tengréla	888	89.19	OQ341215.1 *T. theileri*	Ecuador
* T.theileri_Ten_M_995_RNA*	OR973772	Méré/Tengréla	937	98.79	OQ341205.1 *T. theileri*	Ecuador
* T.theileri_Ten_M_1003_RNA*	OR973773	Méré/Tengréla	925	96.95	AB569249.1 *T. theileri*	Japan
* T.theileri_Ten_Z_1017_RNA*	OR973774	Zébu/Tengréla	1048	97.97	OQ341206.1 *T. theileri*	Ecuador
* T.theileri_Ten_M_1023_RNA*	OR973775	Méré/Tengréla	940	97.12	AB569249.1 *T. theileri*	Japan
* T.theileri_Ten_M_1029_RNA* ^8^	OR973776	Méré/Tengréla	1168	91.27	KY412803.1 *T. theileri*	Austria
		gGAPDH				
*T.theileri_Kor_M_9_GAPDH* **^2^**	OR966684	Méré/Korhogo	865	97.38	XM_029023637.1 *T. theileri*	United Kingdom
*T.vivax_Diko_N_225_GAPDH* **^3^**	OR966685	N’Dama/Dikodougou	752	90.83	MK674038.1 *T. vivax*	Cameroon
*T.vivax_Ferk_M_390_GAPDH*	OR966686	Méré/Ferkéssédougou	424	91.23	MK674038.1 *T. vivax*	Cameroon
*T.vivax_Ferk_M_395_GAPDH*	OR966687	Méré/Ferkéssédougou	391	89.88	MK674038.1 *T. vivax*	Cameroon
*T.theileri_Ouan_B_617_GAPDH* **^4^**	OR966688	Baoulé/Ouangolodougou	342	93.08	XM_029023637.1 *T. theileri*	United Kingdom
*T.vivax_Ouan_M_691_GAPDH*	OR966689	Méré/Ouangolodougou	331	84.72	MK674007.1 *T. vivax*	Cameroon
*T.vivax_Ouan_M_696_GAPDH*	OR966690	Méré/Ouangolodougou	339	84.55	MK674007.1 *T. vivax*	Cameroon
*T.vivax_Boun_M_853_GAPDH*	OR966691	Méré/Boundiali	317	85.96	MK674007.1 *T. vivax*	Cameroon
*T.theileri_Ten_M_935_GAPDH* ^7^	OR966692	Méré/Tengréla	349	95.13	XM_029023637.1 *T. theileri*	United Kingdom
*T.theileri_Kor_M_42_GAPDH* **^5^**	OR966693	Méré/Korhogo	504	97.35	XM_029023637.1 *T. theileri*	United Kingdom
*T.theileri_Kor_M_43_GAPDH* **^6^**	OR966694	Méré/Korhogo	294	97.29	XM_029023637.1 *T. theileri*	United Kingdom
*T.theileri_Sin_N_161_GAPDH*	OR966695	N’Dama/Sinématiali	323	89.30	LC618038.1 *T. theileri*	Japan
*T.theileri_Diko_N_231_GAPDH*	OR966696	N’Dama/Dikodougou	245	88.36	HQ664784.1 *T. theileri*	Brazil
*T.congolense_Mben_M_271_GAPDH*	OR966697	Méré/M’Bengué	270	90.08	AJ620289.1 *T. congolense*	Cameroon
*T.theileri_Mben_M_272_GAPDH*	OR966698	Méré/M’Bengué	303	96.08	AJ620282.1 *T. theileri*	Cameroon
*T.vivax_Ferk_M_382_GAPDH*	OR966699	Méré/Ferkéssédougou	300	91.04	MK674044.1 *T. vivax*	Cameroon
*T.theileri_Ouan_B_617_GAPDH*	OR966700	Baoulé/Ouangolodougou	337	93.31	XM_029023637.1 *T. theileri*	United Kingdom
*T.vivax_Boun_N_816_GAPDH*	OR966701	N’Dama/Boundiali	481	88.12	MK674044.1 *T. vivax*	Cameroon
*T.theileri_Ten_M_935_GAPDH* **^7^**	OR966702	Méré/Tengréla	338	97.98	LC618038.1 *T. theileri*	Japan
*T.theileri_Ten_M_1029_GAPDH* **^8^**	OR966703	Méré/Tengréla	328	92.76	XM_029023637.1 *T. theileri*	United Kingdom

ITS1: internal transcribed spacer 1, ITS2: internal transcribed spacer 2, bp: base pair, *T: Trypanosoma*, gGAPDH: glycosomal glyceraldehyde-3-phosphate dehydrogenase gene, ID: identifying; IDs with same letter superscripts labeled in red are cases of mixed infection; IDs with same number superscripts written in blue are cases of species identified by at least two of the primer types in this study.

## Data Availability

All sequences generated in the present study are available in the GenBank database under the accession numbers in Table 1.

## Supplementary Materials

The following supporting information can be downloaded at: https://www.mdpi.com/article/10.3390/pathogens13030262/s1, Table S1: Trypanosomes band sizes expected and primers sequences; Figure S1: Cluster W alignment of *T. vivax* strain based on Internal Transcribed Spacer 1 (ITS1); Figure S2. Cluster W alignment of *T. theileri* strain according to the Glycosomal glyceraldehyde-3-phosphate dehydrogenase gene (gGAPDH); Figure S3. Cluster W alignment of *T. congolense* strain according to partial 18S, ITS1, 5.8S, ITS2 and partial 28S rRNA genes.
