# Supplementary material for: Genetic Diversity of Trypanosomes Infesting Cattle from Savannah District in North of Côte d’Ivoire Using Conserved Genomic Signatures: rRNA, ITS1 and gGAPDH
Source: Pathogens. 2024 Mar 19;13(3):262. doi: 10.3390/pathogens13030262 (PMC10974534; doi:10.3390/pathogens13030262)
Supplement: Supplementary file 1 [file pathogens-13-00262-s001.zip › pathogens-2885881-supplementary.pdf]

Table S1. Trypanosomes band sizes expected and primers sequences.

PCR DNA sequence target	Primer sequence (5′-3′)	Trypanosomes species and band sizes	References
ITS1 PCR, target non-coding DNA between 18S and 5.8S rRNA genes	ITS1 CF: 5′-CCGGAAGTTCACCGATATTG-3′<br>ITS1 BR: 5′-TTGCTGCGTTCTTCAACGAA-3′	*T.vi (250bp); T.br (480bp); T.cs (700bp); T.cf (710bp); T.s (400bp); T.s.t (370bp); T.th (273bp)*	[11]
Nested ITS PCR target partial 18S, ITS1, 5.8S, ITS2 and partial 28S rRNA genes	ITS1: 5′- GATTACGTCCCTGCCATTTG-3′<br>ITS2: 5′-TTGTTCGCTATCGGTCTTCC-3′<br>ITS3: 5′-GGAAGCAAAAGTCGTAACAAGG-3′<br>ITS4: 5′-TGTTTTCTTTTCCTCCGCTG-3′	*T.vi (620bp); T.br (1215bp); T.cs (1408-1501bp); T.cf (1408-1501bp); T.s (847bp); T.s.t (951bp); T.th (998bp)*	[19]
Nested PCR target Glycosomal glyceraldehyde-3-phosphate dehydrogenase gene (gGAPDH)	G3: 5′-TTYGCCGYATYGGYCGCATGG-3′<br>G5: 5′-ACMAGRTCCACCACRCGGTG-3′<br>G1: 5′-CGCGGATCCASGGYCTYMTCGGBAMKGAGAT-3′<br>G4a: 5′-GTTYTGCAGSGTCGCCTTGG-3′<br>G4b: 5′-CCAMGASACVAYCTTGAAGAA-3′<br>Gs: 5′-GCSTAY CAG ATG AAG TACGAC-3′	*T.vi (620bp); T.br (1215bp); T.cs (1408-1501bp); T.cf (1408-1501bp); T.s (847bp); T.s.t (951bp); T.th (998bp)*	[20]

PCR: Polymerase chain reaction; ITS: Internal Tanscribed Space; DNA: Desoxyribo Nucleic Acid.

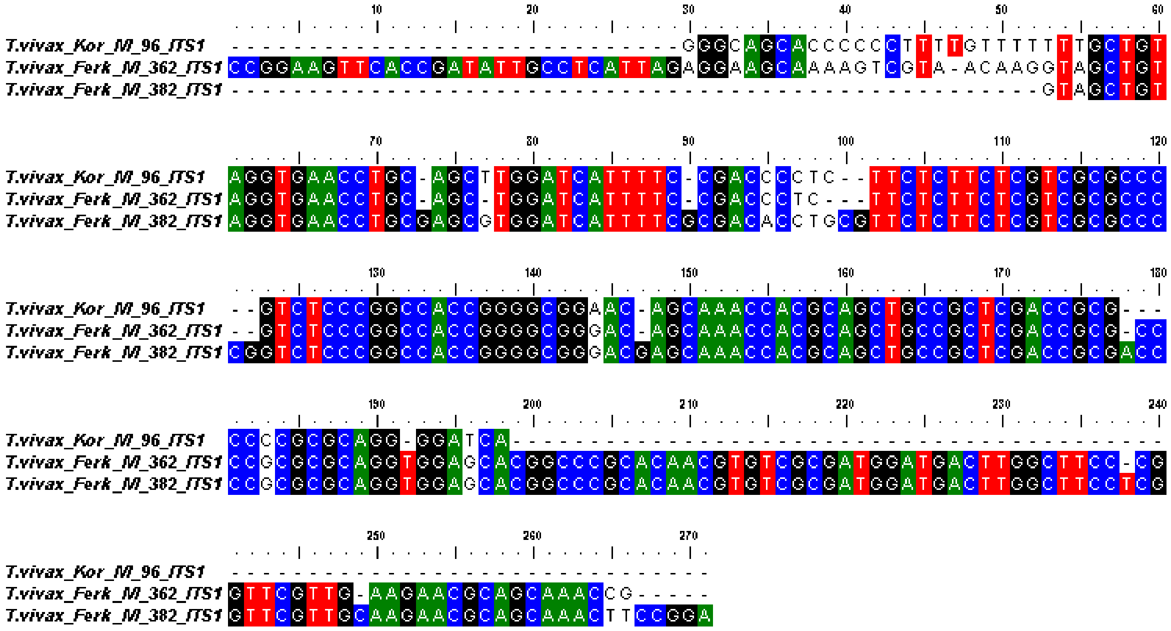

Figure S1. Cluster W alignment of T. vivax strain based on Internal Transcribed Spacer 1 (ITS1).

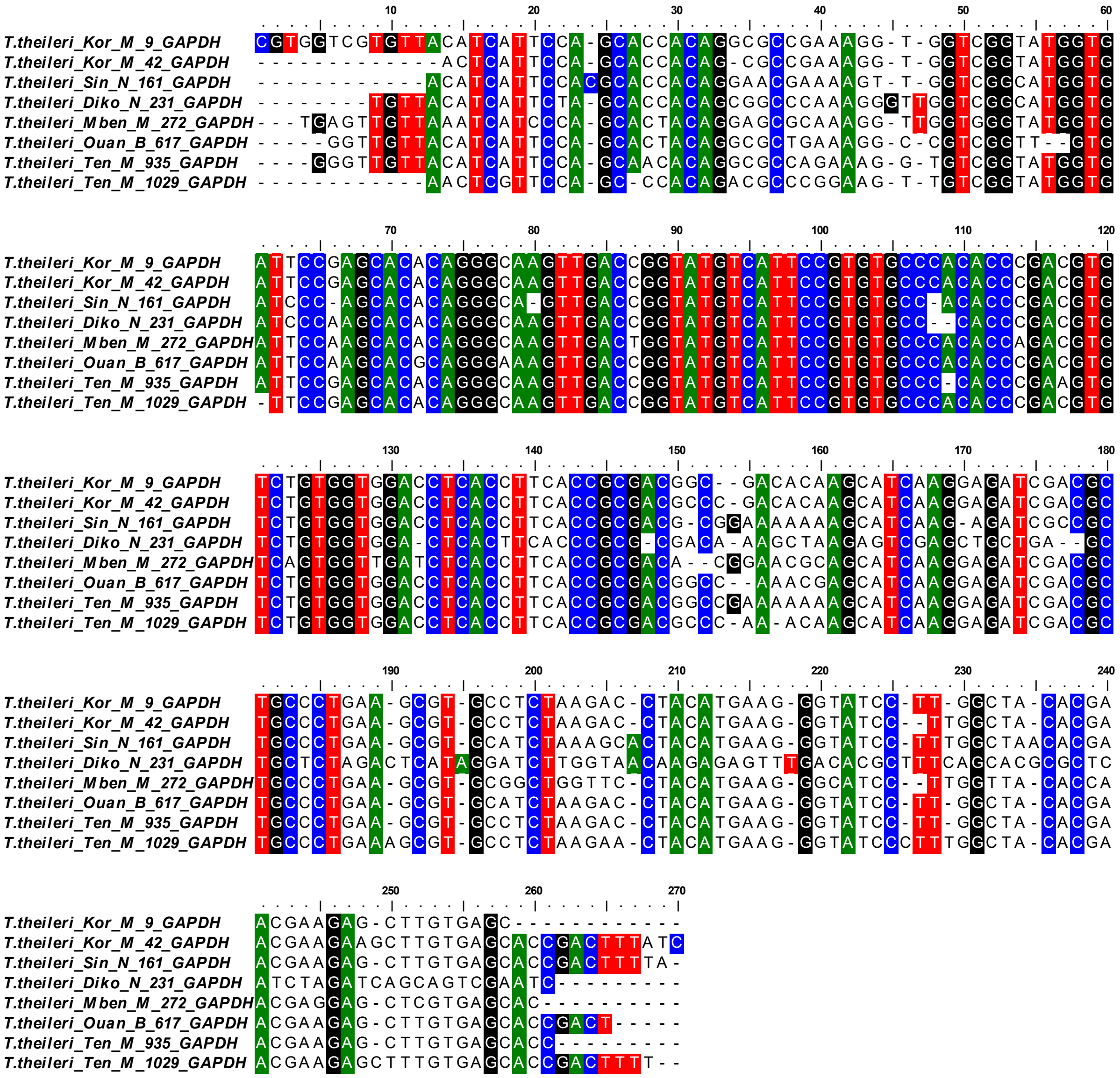

Figure S2. Cluster W alignment of *T. theileri* strain according to the Glycosomal glyceraldehyde-3-phosphate dehydrogenase gene (gGAPDH).

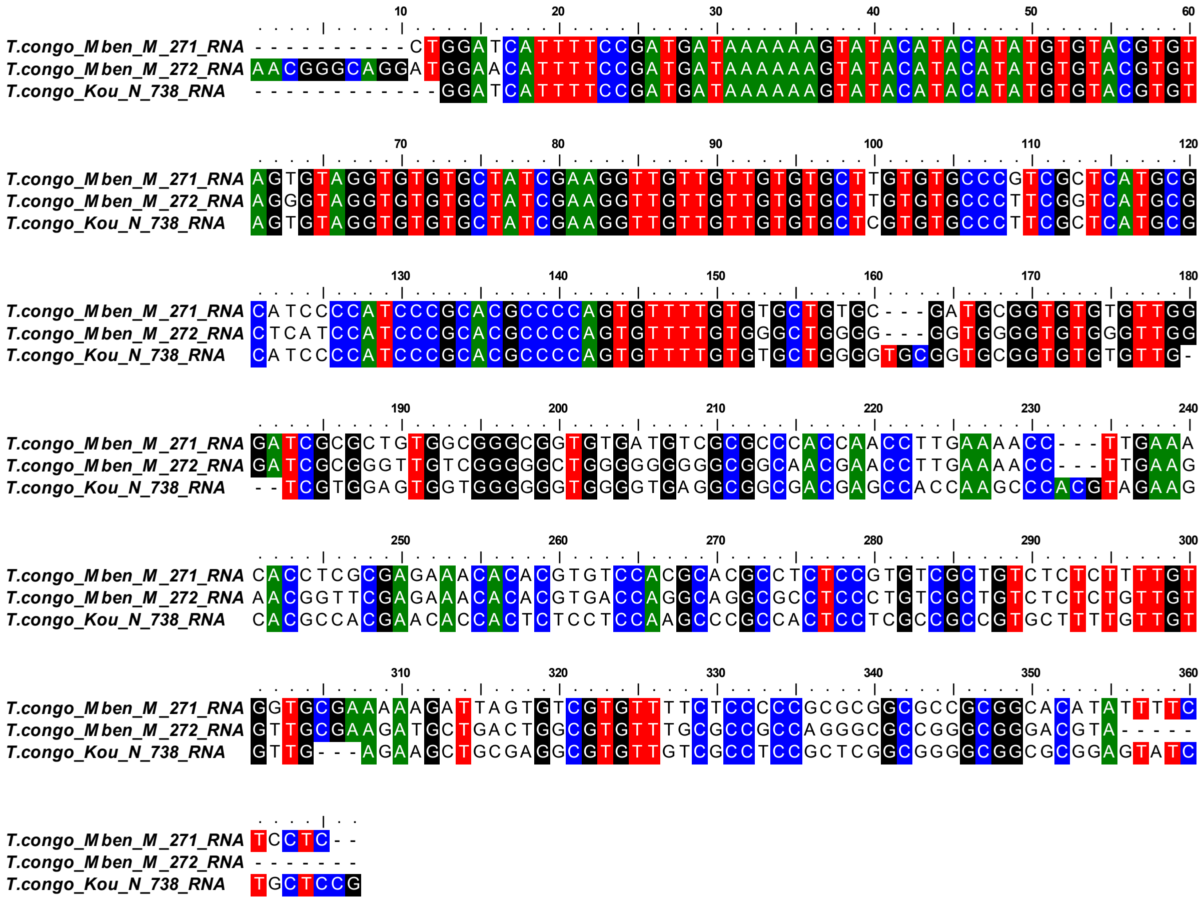

Figure S3. Cluster W alignment of *T. congolense* strain according to partial 18S, ITS1, 5.8S, ITS2 and partial 28S rRNA genes.
